# Narrative Review of Biomarkers in Patients with Peri-implantitis

**DOI:** 10.31661/gmj.v13iSP1.3556

**Published:** 2024-12-30

**Authors:** Soroush Ghodratizadeh, Naghmeh Shenasa, Omid Tavakol, Mehdi Mohamadinia, Hossein Gandomkar, Mohammadreza Behnam Roudsari, Khayrolnesa Sadighi

**Affiliations:** ^1^ Istanbul Aydin University, Faculty of Dentistry, Istanbul, Turkey; ^2^ Private Practice, Formerly affiliated with Shahrekord University of Medical Science, Endodontics Department, Shahrekord, Iran; ^3^ Prosthodontist, Private Practice, Shiraz, Iran; ^4^ Department of Dental Prosthesis, School of Dentistry, Ahvaz Jundishapur University of Medical Sciences, Ahvaz, Iran; ^5^ Department of Surgical Oncology, Tehran University of Medical Medicine, Tehran, Iran; ^6^ Dental Research Center, Research Institute of Dental Sciences, School of Dentistry, Shahid Beheshti University of Medical Sciences, Tehran, Iran; ^7^ Department of Periodontics, Mashhad University of Medical Science, Mashhad, Iran

**Keywords:** Peri-implantitis, Biomarkers, Review, Oxidative Stress, MicroRNAs

## Abstract

**Background:**

Peri-implantitis is caused by the breakdown of homeostasis between the host’s response to microbial pathogens. The aim of this study was to assess clinical studies by use of a systematic review on some mouth biomarkers except of interleukin, active metalloproteinase (MMP) and TNF-α in peri-implantitis patients.

**Materials and Methods:**

A regular and complete search was conducted through mesh keywords by search of the Science Direct, PubMed, Google Scholar database until August 6, 2024. Those articles that reported biomarkers other than interleukin, MMP and TNF-α were included in this review. The outcome was defined to be peri-implantitis. Two reviewers have searched and screened the articles completely independently of each other. For assessing the quality of the studies, risk of bias tool developed by Downes et al. were used.

**Results:**

In general, 41 articles were found for this review. Based on our findings, key markers include Neutrophil extracellular traps (NETs), proinflammatory cytokines, oxidative stress markers, salivary biomarkers, microRNAs, extracellular vesicles, proteomic and metabolomic changes, and microbial markers. Stress markers like cortisol also play a role. Risk of bias is low in most studies.

**Conclusion:**

Biomarkers found in this study suggest a complex cascade of events involved in pathophysiological pathway of peri-implantitis including the microbial colonization, immune activation, bone resorption, oxidative stress, vascular changes, stress responses, and epigenetic modifications.

## Introduction

In line with the increase in the use of implants at the level of human societies, the number of cases of peri-implantitis will increase as a result. Peri-implantitis is an inflammatory disease that leads to inflammation and loss of soft and hard tissue [1]. Peri-implantitis is caused by the breakdown of homeostasis between the host’s response to microbial pathogens [1][2]. On the other hand, a reversible inflammation caused by plaque called mucositis is formed around the implant, which shows itself along with redness, swelling and bleeding [2]. If peri-implant mucositis is not treated or is inadequately treated, peri-implantitis can develop [2].

Correct diagnosis and effective follow-up of the patient after dental implant implantation is of particular importance [3]. For the diagnosis of peri-implantitis, medical science emphasizes and pays attention to clinical and radiographic evaluation, while this diagnostic evaluation does not have a high sensitivity to diagnose the early stages of the disease. Confirmatory clinical considerations which there are besides of implants often influenced by the prosthesis, while the detection of the marginal bone surface on periapical radiographs may be helpful. Therefore, currently, it can be said that non-invasive and reliable diagnostic tools can lead to a better diagnosis process in the early stages of peri-implantitis and start a faster treatment for the patient in question [2][4][5].

There are biomarkers in saliva that can be used as a non-invasive, easy and low-cost method for early diagnosis of oral diseases [6]. Preventing the early progression of periodontal diseases by using biomarkers in a targeted way is increasing. Biomarkers are biological indicators with a high prognosis and predictive ability that can indicate the onset or development of a pathology well. These indicators should be easy, accurate and fast to measure. The applications of biomarkers in health and prediction in the diagnosis of diseases are of great interest [7].

Evaluating any relationship between biomarkers to determine and follow up the reaction in the face of peri-implantitis may lead to reopening a path that will ultimately play a role in preventing and stopping the host’s inflammatory response against microorganisms, so a unique approach can be designed for each patient. However, different and very diverse results are seen in numerous studies that have been conducted in this field [1]. The aim of this study was to review on clinical studies on some mouth biomarkers except of interleukin, active metalloproteinase (MMP) and TNF-α in peri-implantitis patients and thereafter report the results with implications for clinical application.

## Material and Methods

**Table:** Table1. Characteristics of Included Studies

**First author**	**year**	**Sample size**	**Patients**	**Biomarkers**	**Outcome**	**Main finding**
Al-Bakri[10]	2024	64	Samples from patients with peri-implantitis, periodontitis, and controls	Neutrophil extracellular traps (NETs)	To measure NETs in tissue samples	Neutrophils and connective tissue damage were more evident in peri-implantitis; NET markers were higher in mucosal samples of peri-implantitis.
Al-Sowygh[11]	2018	79	T2DM patients (WS and NS) and healthy individuals (WS and NS)	Soft tissue inflammatory markers and CBL	Assessing peri-implant inflammation and CBL	Similar inflammation in WS and NS with T2DM; higher inflammation in WS than NS without T2DM.
Alasqah[12]	2019	50	Obese and non-obese patients	Plaque index, bleeding on probing, probing depth, CAL, CBL	To compare peri-implant indicators in obese vs. non-obese	Obese patients showed worsened peri-implant parameters and elevated inflammatory biomarkers.
Aldulaijan[13]	2022	96		Salivary alpha amylase (AA) and mucin-4 levels	Examining AA and mucin-4 pre- and post-PM treatment	No significant change in salivary AA and mucin levels after PM.
Algohar[14]	2020	60	Groups: healthy, peri-implant mucositis, peri-implantitis	Procalcitonin in saliva and PICF	Evaluate procalcitonin levels in healthy and diseased patients	Higher procalcitonin in diseased patients, correlating with clinical signs of inflammation.
Alresayes[15]	2021	88	Patients with and without peri-implantitis in two groups	Cortisol levels in peri-implant sulcular fluid (PISF)	Investigate cortisol levels in PISF for peri-implantitis diagnosis	Inconclusive findings on cortisol level variations in peri-implantitis. More research needed for PISF cortisol’s diagnostic role.
Alsahhaf[16]	2023	94	30 with peri-implantitis, 32 with mucositis, 32 healthy	Biomarkers: CCL-20, BAF, RANK-L, OPG	Evaluate novel biomarkers in peri-implantitis	Increased inflammatory markers in peri-implant disease; poor probing depth and bleeding observed in affected patients.
Chaparro[17]	2021	54	Healthy, peri-implant mucositis, and peri-implantitis patients	microRNA-21-3p, microRNA-150-5p, extracellular vesicles (EVs)	Diagnostic potential of miRNA and EVs in peri-implant diseases	Higher EVs and reduced miRNA levels in peri-implantitis, indicating disease progression potential.
Chaparro[18]	2022	19	Dental implant patients: healthy, mucositis, or peri-implantitis	CCL-20/MIP-3α, BAFF/BLyS, RANKL, OPG	Investigate biomarker concentrations in PICF	RANKL potentially key in peri-implantitis development; further study of BAFF/BLyS suggested for early diagnosis.
Chaparro[19]	2020	54	21 peri-implantitis implants, 24 healthy implants	DNA methylation related to titanium presence	Analyze methylation patterns and titanium levels in peri-implantitis	Increased methylated DNA and titanium linked in peri-implantitis, suggesting possible influence of titanium dissolution.
Daubert[20]	2019	44	21 peri-implantitis and 24 healthy implants	DNA Methylation to Titanium	Analyze global methylation and titanium levels in peri-implantitis	Increased methylation found in peri-implantitis, suggesting titanium may affect methylation independently.
de Mello-Neto[21]	2021	47	27 with mucositis and 20 with peri-implantitis	CSF-1, S100A8/A9, S100A12 in saliva	Assess peri-implant treatment’s effect on saliva biomarkers	Treatment improved clinical outcomes and lowered CSF-1 and S100A8/A9; no correlation found with PICF levels.
Dewan[22]	2023	60	20 smokers, 20 non-smokers with peri-implantitis, 20 non-smokers without	suPAR	Evaluate suPAR levels in smokers vs. non-smokers with/without peri-implantitis	suPAR levels were correlated with peri-implant probing depth in non-smokers.
Drafta[23]	2021	10	7 with implants and 3 fully dentate individuals	Antioxidant status (TAS), salivary lactate dehydrogenase (LDH)	Assess TAS, LDH and their link to peri-implant bone loss	TAS and cytokines in saliva may be related to bone loss risk over time in implants.
Esberg[24]	2019	25	25 peri-implantitis sites	Proteomic profile	Identify PICF protein patterns linked to peri-implantitis	Specific PICF proteomic patterns were linked to active peri-implantitis and implant loss, with 52 proteins implicated.
Figueiredo[25]	2020	20	Group with peri-implantitis (PI group, n=20)	TIMP-1 and TIMP-2 in gingival tissue	Assess immune-inflammatory markers in tissues of periodontal vs. peri-implant diseases	Biomarkers were similar between groups, with no notable difference in metalloproteinase inhibitors.
Flores[26]	2022	13	15 soft tissue and 6 bone tissue samples from 13 peri-implantitis patients	APRIL, BAFF, Osteonectin, α-SMA in tissue	Characterize soft and bone tissue changes in peri-implantitis	APRIL and BAFF linked to bone resorption; low osteonectin may impair bone remodeling.
Gürlek[27]	2017	97	Samples from healthy, mucositis, and peri-implantitis conditions in 97 implants/teeth	sRANKL, OPG, Albumin in GCF/PICF	Examine cytokine levels and bacterial presence in GCF/PICF	Elevated sRANKL in gingivitis vs. mucositis; similar biomarker profiles in peri-implantitis and periodontitis.
Jansson[28]	2021	163	Implant sites (healthy and diseased) after 10+ years	Treg cytokines, IFN proteins	Explore cytokine profiles at periodontitis, peri-implantitis, and healthy sites	Intra-individual cytokine profiles matched for peri-implantitis and periodontitis, differing only between tooth and implant sites.
Lira-Junior[29]	2020	43	43 patients, including those with mucositis (20) and peri-implantitis (23)	CSF-1 in saliva and PICF	Analyze CSF-1 levels across saliva and PICF in peri-implant diseases	Higher CSF-1 in PICF for peri-implantitis than mucositis; no significant difference in salivary CSF-1 and IL-34.
López-Jornet[30]	2024	160	160 patients in 4 groups: healthy, maintenance, implants, and maintenance with implants	Oxidative stress biomarkers: FRAP, TEAC, CUPRAC, AOPP, TP	Evaluate stress biomarkers in implant patients	No significant differences in oxidative stress biomarker levels between implant and non-implant groups.
Marcello-Machado [31]	2020	16	Edentulous patients with narrow diameter implants (NDI)	Cytokine release in PICF	Track cytokine patterns and NDI success factors	Implant stability improved; success affected by smoking, plaque, and gingival indices.
Marques Filho[32]	2018	42	Groups with and without peri-implantitis	Cytokines MCP-1, MIP-1α, MIP-1β, and herpesvirus	Measure cytokine and herpesvirus levels in peri-implantitis	Herpesvirus levels 1.97 times higher in peri-implantitis; MIP-1β significant in peri-implant group.
Menini[33]	2021	14	PICF from peri-implantitis and control groups	MiRNAs linked to bone resorption	Compare miRNA expression in bone resorption cases	MiRNAs show potential for non-invasive bone resorption diagnosis in PICF samples.
Mousavi Jazi[34]	2015	31	PICF from 50 implants	Oxidative stress markers: MDA, SOD, TAC	Identify oxidative stress differences in PICF	PPD linked to MDA and TAC; oxidative markers not diagnostic for peri-implant disease.
Pallos[35]	2022	42	Peri-implant sites in healthy and peri-implantitis patients	Salivary microbiome diversity	Examine microbiome in peri-implant vs. healthy sites	Distinct microbiome in peri-implantitis; BoP affects microbial diversity.
Priyadharsini[36]	2024	40	Groups with varying peri-implant conditions	C-reactive protein (CRP)	Compare CRP in peri-implant health and disease	CRP increased with peri-implantitis severity; highest in advanced disease.
Rakic[37]	2015	369	Patients with and without peri-implantitis	Genetic marker CD14-159 C/T, RANKL, OPG	Identify genetic risk factors for peri-implantitis	CD14-159 C/T linked to peri-implantitis risk; potential biomarker.
Ramenzoni [38]	2021	20	Patients with periodontitis vs. healthy controls	Lactoferrin in gingival pockets	Use lactoferrin to assess inflammation in periodontitis	Elevated lactoferrin in periodontitis; potential inflammation indicator.
Renvert[39]	2015	41	Peri-implantitis cases without treatment	VEGF in crevicular fluid	Assess inflammatory markers in untreated peri-implantitis	Higher VEGF in severe inflammation; potential indicator of disease progression.
Saito[40]	2024	76	Healthy, mucositis, and peri-implantitis patients	Endothelin-1 (ET-1)	Examine ET-1 in peri-implant disease progression	Increased ET-1 in mucositis; potential for early detection of implant inflammation.
Sanchez-Siles[41]	2016	70	Healthy and peri-implantitis patients	Salivary oxidative stress markers	Compare stress levels in peri-implantitis vs. controls	No difference in oxidative stress markers between peri-implantitis and controls.
Sharma[42]	2024	100	Peri-implantitis patients vs. healthy controls	C-reactive protein (CRP)	Assess CRP in peri-implant vs. control groups	Higher CRP in peri-implantitis than controls; shows inflammation severity.
Shelke[43]	2020	66	Groups with healthy, mucositis, and peri-implantitis	Periostin in peri-implant sulcular fluid (PISF)	Compare periostin levels across peri-implant conditions	Elevated periostin in disease states; useful for early detection of peri-implantitis.
Song[44]	2019	40	Patients with peri-implantitis and healthy controls	hs-CRP, SOD, GSH-Px, MDA in GCF	Analyze inflammatory markers in GCF and peri-implantitis	Increased markers in peri-implantitis; correlated with probing depth and bleeding index.
Soysal[45]	2024	50	Peri-implantitis and healthy implants	IFNα, GRα, sAA gene expression	Study cytokine and stress-related gene markers	sAA higher in stressed peri-implantitis; GRα lower but not significantly.
Teixeira[46]	2020	77	Gingivitis, periodontitis, mucositis, peri-implantitis	sTREM-1, PGLYRP1, TIMP-1	Examine sTREM-1 axis in peri-implant disease	Markers linked to inflammation; potential for identifying implant inflammation.
Urvasizoglu[47]	2021	8	Peri-implantitis vs. healthy implant patients	MicroRNA in saliva samples	Profile miRNA for peri-implantitis detection	miR-4484 potential early diagnostic marker for peri-implantitis.
Urvasizoglu[48]	2023	45	Peri-implantitis vs. non-affected patients	CXCL9, CXCL12, CXCL14	Identify molecular markers for peri-implantitis progression	CXCL14 and miR-4484 found to be potential early biomarkers.
Wang[49]	2016	68	Patients with healthy and peri-implantitis implants	VEGF, TIMP-2, OPG in PICF	Measure inflammation markers in PICF	Increased TIMP-2, VEGF, and OPG in peri-implantitis; potential predictive markers.
Ustaoğlu[9]	2023	60	Peri-implantitis vs. healthy controls	Oxidative stress markers: TAC, TOC, OSI, ARE	Assess oxidant-antioxidant balance in peri-implantitis	Higher TOC, lower TAC and ARE; KMW important for antioxidant defense.

### Search Method

A regular and comprehensive search was conducted through mesh keywords; these keywords included biomarkers, peri-implantitis. Two reviewers performed an unrestricted search of the Science Direct, PubMed, Google Scholar database until August 6, 2024. Reportable items for this study were reviewed based on Prism, and an overview of the results of those studies is reported in this review.

### Eligibility Criteria

Those articles that reported biomarkers other than interleukin, MMP and TNF-α were included in this review. The outcome was defined to be peri-implantitis. Two reviewers have searched and screened the articles completely independently of each other. In the case of disagreements in the results obtained in each of the screening stages, the opinion of the third reviewer has been taken into account, or in case of disagreement, it has been resolved through two-way discussion. The final decision was made regarding the choice of that decision.

### The Quality of the Articles

Assessing the risk of bias is a key step in conducting any review study. It can provide appropriate information about each of the decision-making steps in the implementation of a regular review, and it plays a very important role in the final evaluation of the strength of the evidence. There are several tools for assessing the risk of bias. In this study, the method and tools developed by Downes et al. [8] were used. This tool is prepared by relevant experts based on Delphi methodology, which can be used for cross-sectional studies. The components of this tool are based on a combination of evidence, epidemiological processes, the experiences of researchers and participants in the Delphi process.

For each question in this tool, the articles were evaluated and if they met those criteria, the answer was yes, or if they didn’t have that criterion, a no answer was used, and if it was unknown, then the answer was used by unknown (Table-1). Low, medium, and high degree of biases were determined, although no grading criteria was provided by the developers of this tool. Two reviewers independently evaluated the quality of the articles using this tool. In case of disagreement, they discussed between them or the third reviewer have the final opinion.

## Results

**Figure-1:**
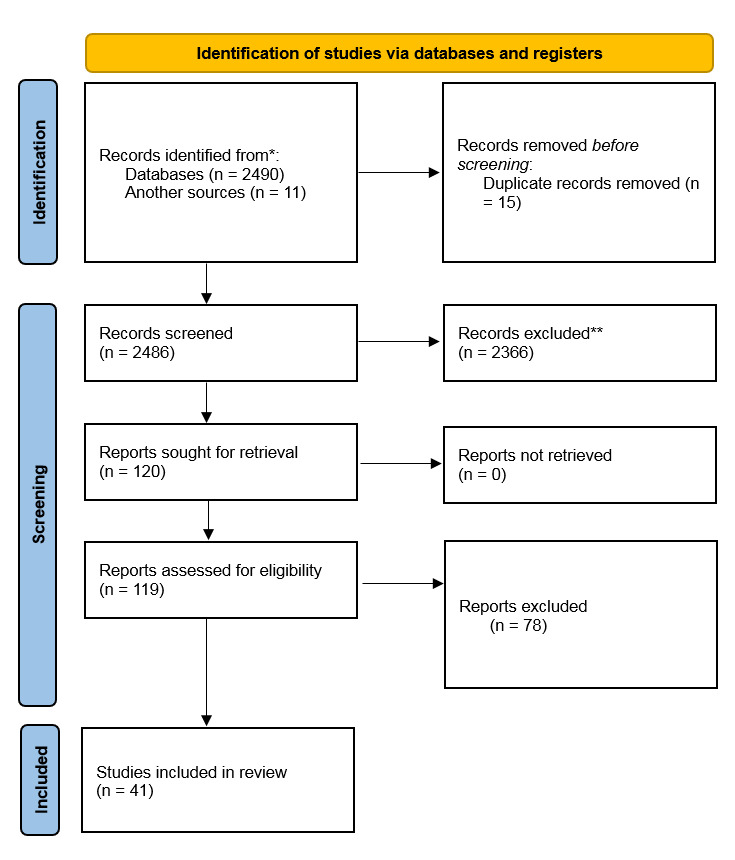
Figure 1. PRISMA flow-chart depicted.

**Table:** Table2. Categories of Biomarkers of Peri-implantitis

**Category**	**Marker**	**References**
**Cytokines and Chemokines**	Monocyte Chemoattractant Protein-1 (MCP-1)	Marques Filho et al. [34]
**Cytokines and Chemokines**	Macrophage Inflammatory Protein-1α (MIP-1α) and MIP-1β	Marques Filho et al. [34]
**Cytokines and Chemokines**	CCL-20	Alsahhaf et al. [23]
**Cytokines and Chemokines**	RANKL (Receptor Activator of Nuclear Factor κ-B Ligand)	Alresayes et al. [22], Chaparro et al. [24][25], Dewan et al. [28]
**Cytokines and Chemokines**	BAFF (B-Cell Activating Factor)	Alresayes et al. [22], Chaparro et al.[(24, 25], Flores et al. [13]
**Cytokines and Chemokines**	OPG (Osteoprotegerin)	Alsahhaf et al. [23], Chaparro et al. [24][25], Dewan et al. (28)
**Cytokines and Chemokines**	sRANKL (Soluble RANKL)	Gürlek et al. [30]
**Cytokines and Chemokines**	sTREM-1 (Soluble Triggering Receptor Expressed on Myeloid Cells-1)	Teixeira et al. [46]
**Cytokines and Chemokines**	PGLYRP-1 (Peptidoglycan Recognition Protein 1)	Teixeira et al. [46]
**Cytokines and Chemokines**	TIMP-1 and TIMP-2 (Tissue Inhibitor of Metalloproteinases)	Figueiredo et al. [12], Wang et al. [48]
**Cytokines and Chemokines**	CSF-1 (Colony-Stimulating Factor 1)	de Mello-Neto et al. [27], Lira-Junior [32]
**Cytokines and Chemokines**	VEGF (Vascular Endothelial Growth Factor)	Renvert et al. [39], Wang et al. [48]
**Cytokines and Chemokines**	APRIL (A Proliferation-Inducing Ligand)	Flores et al. [13]
**Proteins and Growth Factors**	Endothelin-1 (ET-1)	Saito et al. [40]
**Proteins and Growth Factors**	Periostin	Shelke et al. [43]
**Oxidative Stress Markers**	MDA (Malondialdehyde)	Mousavi Jazi et al. [35], Song et al. [44]
**Oxidative Stress Markers**	TAC (Total Antioxidant Capacity)	Mousavi Jazi et al. (35), Song et al. [44]
**Oxidative Stress Markers**	SOD (Superoxide Dismutase)	Song et al. [44]
**Oxidative Stress Markers**	GSH-Px (Glutathione Peroxidase)	Song et al. [44]
**Oxidative Stress Markers**	Salivary Total Antioxidant Status (TAS)	Drafta et al. [10], López-Jornet [33]
**MicroRNAs (miRNAs)**	miRNA-21-3p	Chaparro et al. [24]
**MicroRNAs (miRNAs)**	miRNA-150-5p	Chaparro et al. [24]
**MicroRNAs (miRNAs)**	miR-4484	Urvasizoglu et al. [17][47]
**MicroRNAs (miRNAs)**	miRNAs as Biomarkers for Bone Resorption	Menini et al. [15]
**Cortisol and Stress Markers**	Cortisol in Peri-Implant Sulcular Fluid (PISF)	Alresayes et al. [22]
**Cortisol and Stress Markers**	Salivary Alpha Amylase (sAA)	Aldulaijan [29], Soysal et al. [45]
**Cortisol and Stress Markers**	Glucocorticoid Receptor-Alpha (GRalpha)	Soysal et al. [45]
**Extracellular Vesicles (EVs) and DNA Methylation**	Extracellular Vesicles (EVs)	Chaparro et al. [24]
**Extracellular Vesicles (EVs) and DNA Methylation**	Methylated DNA Cytosine (5mC)	Daubert et al. [26]
**Proteomic and Metabolomic Markers**	Proteins Linked with Implant Loss	Esberg et al. [11]
**Proteomic and Metabolomic Markers**	Osteonectin	Flores et al. [13]
**Salivary and Crevicular Fluid Markers**	Salivary Biomarkers (CRP, TAS, sAA, MDA, TAC, SOD, GSH-Px)	Aldulaijan [29], Algohar [21], de Mello-Neto [27], López-Jornet [33], Mousavi Jazi [35], Pallos [36], Rakic [38], Urvasizoglu [17], Urvasizoglu [47], Ustaoğlu [49]
**Salivary and Crevicular Fluid Markers**	Peri-Implant Sulcular Fluid (PISF) Biomarkers (CRP, suPAR, ET-1, Periostin, Cortisol, 5mC)	Alresayes et al. [22], Dewan et al. [28], Saito et al. [40], Shelke et al. [43]
**Microbial Markers**	Salivary Microbiome Composition	Pallos et al. [36]
**Herpesviruses**	HSV1, HSV2, EBV, CMV, VZV, HHV6, HHV7, HHV8	Marques Filho et al. [34]
**Other Markers**	Lactoferrin	Ramenzoni et al. [16]
**Other Markers**	CD14-159 C/T Polymorphisms	Rakic et al. [38]

**Table:** Table3. Risk of Bias Assessment (32 Studies are not Included in this Table due to no Important Identified Bias)

**Study**	**Chaparro[18] **	**Drafta[23] **	**Esberg[24] **	**Figueiredo[25] **	**Flores[26] **	**Marcello-Machado[31] **	**Menini[33] **	**Ramenzoni[38] **	**Urvasizoglu[47] **
Clear aims/objectives	Yes	Yes	Yes	Yes	Yes	Yes	Yes	Yes	Yes
Study design appropriate for the stated aim(s)	Yes	Yes	Yes	Yes	Yes	Yes	Yes	Yes	Yes
Sample size justification	No	No	No	No	No	No	No	No	No
Target/reference population clearly defined?	No	Yes	No	Yes	Yes	Yes	Yes	Yes	No
Sample representative of target/reference population	Yes	Yes	Yes	Yes	Yes	Yes	Yes	Yes	Yes
Selection process likely to represent target/reference population	No	No	No	No	No	No	No	No	No
Variables appropriate to study aims	Yes	Yes	Yes	Yes	Yes	Yes	Yes	Yes	Yes
Variables measured correctly and trialled/piloted/published previously	No	Yes	No	Yes	Yes	Yes	Yes	Yes	No
Clear method to determine statistical significance	Yes	Yes	Yes	Yes	No	Yes	Yes	Yes	Unknown
Methods sufficiently described to enable repeat	No	Yes	Yes	Yes	No	No	Yes	Yes	No
Basic data adequately described	Yes	No	No	Yes	No	No	No	Yes	Yes
Results internally consistent	No	Yes	Yes	Yes	Yes	Yes	No	Yes	No
Results for the analyses described in the methods presented	Yes	Yes	Yes	No	Yes	Yes	Yes	Unknown	Yes
Authors’ discussions and conclusions justified by the results	Yes	Yes	Yes	Yes	Yes	No	No	Yes	Yes
Limitations of the study discussed	Yes	Yes	Yes	No	No	Yes	Yes	No	Yes
Funding sources or conflicts of interest	Yes	Yes	Yes	Yes	Yes	Yes	Yes	Yes	Yes
Ethical approval/consent of participants	Yes	Yes	Yes	Yes	Yes	Yes	Yes	Yes	Yes
Overall risk of bias rating	Moderate	Low	Moderate	Moderate	Moderate	Moderate	Low	Moderate	Moderate

Finally, after the qualitative evaluation of the studies, 41 studies were included here after screening the 119 relevant records (Figure-1). In general, 41 articles were found for this review, among which the identified biomarkers were in a very wide range. Except for the biomarkers of interleukins and MMP and TNF-α, other biomarkers of the studies were included in this review.

Based on the checklist designed by the researchers, which can be seen in Table-1, the data of each article was extracted. In this study, there was no need to send emails to the corresponding authors to provide their study data other than what was reported.

With the exception of a few minor disagreements, which were resolved by a third party, excellent agreement was reached between the two reviewers for evaluating and screening the articles.

As shown in Table-1., Al-Bakri et al. [18] in a pilot study indicated that a greater presence and involvement of Neutrophil extracellular traps (NETs) are observed in peri-implantitis patients. Additionally, the destruction of connective tissue has been widely observed in these cases. Furthermore, a significant higher expression of markers related to NETs has been observed in the mucosal peri-implantitis samples compared to the control and periodontitis groups. In a related study, Al-Sowygh et al. [19] found that peri-implant soft tissue inflammatory parameters, including the peri-implant plaque index and probing depth, as well as crestal bone loss, were worse among waterpipe consumers compared to never smokers. This suggests that smoking habits can significantly impact peri-implant health. Similarly, Alasqah et al. [20] compared obese and non-obese patients and found that peri-implant parameters worsened and proinflammatory biomarkers were significantly higher in obese patients. This increase in proinflammatory biomarkers in the crevice fluid around the implant can moderate the inflammation around the implant, highlighting the role of obesity in peri-implantitis. Another study by Alresayes et al. [22] assessed cortisol levels in peri-implant sulcular fluid (PISF) of patients with and without peri-implantitis, but found inconclusive differences. The authors recommend further studies to explore PISF cortisol's diagnostic potential for peri-implantitis.

In a study by Alsahhaf et al. [23], the levels of biomarkers CCL-20, BAF, RANK-L, and OPG were determined, and these biomarkers were found to have high levels in peri-implant crevicular fluid (PICF) in the studied patients. This finding aligns with the results from Al-Bakri et al., suggesting a common inflammatory pathway in peri-implantitis. Chaparro et al. [24] further explored this pathway, finding an increased concentration of extracellular vesicles (EVs) and a downregulated expression of miRNA-21-3p and miRNA-150-5p associated with the development of peri-implantitis. These findings were complemented by Chaparro et al. [9], who proposed that RANKL could shed light on the pathogenesis involved in the transition from peri-implant health to peri-implantitis. Additional research on BAFF/BLyS is needed for early peri-implantitis diagnosis.

Chaparro et al. [25] extended this research, concluding that patients with peri-implantitis show an upregulation of the RANKL/BAFF-BLyS axis, a finding that requires further investigation in studies with a larger sample size. Daubert et al. [26] added to this body of research by finding higher levels of methylated DNA cytosine (5mC) in peri-implantitis cases compared to controls, with titanium concentrations linked to overall methylation regardless of disease status. These findings highlight the need for further research to clarify whether these associations are causal or not. In a study by de Mello-Neto et al. [27], the effects of peri-implant treatment on salivary levels of CSF-1, S100A8/A9, and S100A12 were examined. The treatment significantly improved clinical outcomes and lowered salivary CSF-1 and S100A8/A9 levels, but these salivary markers did not correlate with their levels in PICF. Dewan et al. [28] conducted a study in 2023, finding that PISF suPAR levels in non-smokers were associated with peri-implant probing depth (PD). This suggests that suPAR could be a useful marker for monitoring peri-implantitis progression. Drafta et al. [10] also contributed to the field, suggesting that salivary total antioxidant status (TAS) and proinflammatory cytokines may be linked to an increased risk of peri-implant bone loss over time. This aligns with the findings of Esberg et al. [11], who identified a proteomic profile linked with implant loss and found 52 specific proteins associated with this outcome. Figueiredo et al. [12] reported no significant differences in TIMP-1 and -2 levels between peri-implantitis and healthy groups, while Flores et al. (13) examined tissue markers and found that APRIL and BAFF may contribute to peri-implant bone resorption, while lower osteonectin levels might be related to impaired bone remodeling.

Aldulaijan [29] found no change in salivary alpha amylase (AA) and mucin-4 levels before and after non-surgical mechanical debridement in patients with peri-implant mucositis, while Gürlek et al. [30] found significantly higher sRANKL levels in the gingivitis group compared to mucositis, with similar biomarker levels in peri-implantitis and periodontitis groups. Jansson et al. [31] found no significant cytokine (including treg cytokines and interferon (IFN) proteins) differences between periodontitis and peri-implantitis sites, but differences between healthy tooth and implant sites. This highlights the importance of distinguishing between different types of oral inflammation. Lira-Junior [32] found that CSF-1 levels were higher in peri-implantitis PICF than in mucositis, with a significant correlation between CSF-1 in both saliva and PICF. This suggests that CSF-1 could be a useful marker for monitoring peri-implantitis. López-Jornet [33] assessed salivary oxidative stress biomarkers in dental implant patients with or without periodontitis, finding no significant differences in biomarker levels between those with controlled periodontal disease and healthy individuals. Marcelo-Machado et al. [14] monitored cytokine patterns in PICF and examined factors affecting narrow diameter implants' success during the first year, finding significant decreases in probing depth (PD) and implant stability quotient (ISQ), with a stable marginal bone and an 81.3% success rate influenced by various clinical factors.

Marques Filho et al. [34] assessed cytokine levels (MCP-1, MIP-1α, MIP-1β) and herpesviruses (HSV1, HSV2, EBV, CMV, VZV, HHV6, HHV7, HHV8) in saliva from individuals with and without peri-implantitis, finding no significant cytokine differences but a 1.97-fold higher herpesvirus presence in peri-implantitis patients, with a significant association between MIP-1β and herpesvirus in the peri-implantitis group. Menini et al. [15] suggested that MiRNAs could serve as biomarkers for peri-implant bone resorption, paving the way for non-invasive, site-specific liquid biopsy using PICF.

Mousavi Jazi et al. [35] found significant correlations between probing pocket depth (PPD) and oxidative stress markers (MDA, TAC), but no significant changes in these markers between peri-implantitis and healthy implants, indicating their limited utility for distinguishing peri-implant health from disease. Pallos et al. [36] analyzed the salivary microbiome in healthy and peri-implantitis sites, finding differences in microbiome composition, with bleeding on probing (BoP) influencing the diversity of the salivary microbiome. Priyadharsini [37] compared C-reactive protein (CRP) levels in peri-implant health and disease, finding higher CRP levels in peri-implantitis, followed bymucositis, and a positive correlation between CRP levels and disease severity.

Rakic et al. [38] studied the association between CD14-159 C/T polymorphisms and peri-implantitis, finding a link with bone resorption markers RANKL and OPG, and suggesting these polymorphisms as potential biomarkers for peri-implantitis. Ramenzoni et al. [16] investigated the source of Lactoferrin in periodontitis patients, finding higher concentrations of Lactoferrin in periodontal pockets compared to other sources. Renvert et al. [39] examined clinical inflammation, VEGF levels, and bacterial counts in implant crevicular fluid samples from untreated peri-implantitis cases, finding that increased bleeding or suppuration was linked to higher VEGF concentrations in the fluid. Saito et al. [40] investigated Endothelin-1 (ET-1) as a potential biomarker for peri-implant diseases, finding that its elevated presence in PISF, particularly in peri-implantitis, could aid in earlier and more accurate diagnosis when combined with traditional examination methods. Sanchez-Siles et al. [41] found that peri-implantitis did not lead to higher oxidative stress marker concentrations in saliva compared to healthy individuals, suggesting that oxidative stress markers may not be reliable indicators for peri-implantitis.

Sharma et al. [42] observed higher mean CRP levels in peri-implantitis patients (0.615 mg/dL) compared to controls (0.201 mg/dL). Shelke et al. [43] identified periostin levels in peri-implant sulcular fluid (PISF) as a promising tool for early diagnosis of peri-implant diseases, which could aid in treatment planning and improve the longevity of dental implants. Song et al. in 2019 [44] analyzed hypersensitive C-reactive protein (hs-CRP), superoxide dismutase (SOD), glutathione peroxidase (GSH-Px), and malondialdehyde (MDA) levels in gingival crevicular fluid (GCF) of peri-implantitis patients, finding that these markers are involved in peri-implantitis and could serve as auxiliary indicators for its evaluation, with clinical indices correlating with GCF volume and hs-CRP levels. Soysal et al. [45] studied the relationship between interferon (IFN)alpha, psychological stress markers, glucocorticoid receptor-alpha (GRalpha), and salivary alpha amylase (sAA) in salivary from healthy implants andperi-implantitis patients, finding significantly higher sAA expression in peri-implantitis patients with high stress levels, while GRalpha expression was lower but not statistically significant. Teixeira et al. [46] investigated the expression of sTREM-1, its ligand PGLYRP-1, and TIMP-1 in peri-implant diseases, finding no significant differences in the sTREM-1/PGLYRP-1 axis between periodontal and peri-implant diseases, suggesting their potential as markers for both conditions. In the study by Urvasizoglu et al. [17] in 2021, saliva microRNA content, particularly miR-4484, was found to be a promising candidate for the early detection ofperi-implantitis. Urvasizoglu et al. [47] proposed that the varying expressions of CXCL14 and miR-4484 in salivary of peri-implantitis patients could serve as biomarkers for early disease detection. Wang et al. in 2016 [48] studied 34 patients with healthy implants and 34 withperi-implantitis, finding that TIMP-2, VEGF, and OPG levels in peri-implant crevicular fluid were significantly higher in peri-implantitis, suggesting these biomarkers could potentially predict peri-implant diseases. Ustaoğlu et al [9] assessed clinical parameters such as probing depth and gingival index, alongside salivary levels of oxidative stress markers, concluding that increased total oxidant capacity and decreased antioxidant activity could predict peri-implantitis development, with adequate keratinized mucosa width being essential for antioxidant production.

Regarding the imported articles, it can be said that the range of sample size of original articles was from 8 to 369 and the articles were published in the range of 2015 to 2024.

The provided list of biomarkers in Table-2 can be integrated into biological theoretical framework that elucidates the complex interactions involved in peri-implant diseases, such as peri-implantitis.

This framework primarily focuses on inflammation and immune response, oxidative stress, microbial interactions, and stress markers. Cytokines and chemokines, such as MCP-1, MIP-1α, MIP-1β, CCL-20, and CINC, play crucial roles in recruiting immune cells to the site of inflammation, while RANKL and BAFF are involved in osteoclast differentiation and B-cell activation, respectively, contributing to bone resorption and immune modulation. Proteins like endothelin-1 and periostin, along with growth factors, influence vascular and tissue remodeling. Oxidative stress markers, including MDA, TAC, SOD, and GSH-Px, indicate the balance between oxidative damage and antioxidant defense mechanisms, which are critical in the pathogenesis of peri-implantitis. MicroRNAs, such as miRNA-21-3p and miRNA-150-5p, regulate gene expression and may serve as biomarkers for bone resorption and disease progression.

Cortisol and stress markers, like salivary alpha amylase and glucocorticoid receptor-alpha, reflect the body's stress response, which can modulate immune function and inflammation. Extracellular vesicles (EVs) and DNA methylation markers, such as methylated DNA cytosine, are involved in intercellular communication and epigenetic regulation, influencing disease development and progression. Proteomic and metabolomic markers, including osteonectin and proteins linked with implant loss, provide insights into the molecular changes associated with peri-implant tissue breakdown. Salivary and peri-implant sulcular fluid biomarkers, such as CRP, TAS, sAA, and ET-1, offer non-invasive means to monitor disease status. Microbial markers, including the salivary microbiome and herpesviruses, highlight the role of microbial communities in disease initiation and progression. Other markers, such as lactoferrin and CD14-159 C/T polymorphisms, further contribute to the understanding of host-microbe interactions and genetic predispositions. This integrated framework provides a holistic view of the biological processes underlying peri-implant diseases, facilitating more targeted diagnostic and therapeutic strategies. Regarding the risk of bias, the results of which can be seen in Table-3, in some of them, bias and the desired items of our tool were mentioned. Finally, 2 of the articles were placed at the low level in terms of risk of bias and 7 of them at the moderate level, and in all others, there were not any potentially sources of bias, so we finally included all those articles in this review (Table-3).

## Discussion

The comprehensive review of the literature on peri-implantitis highlights a multifaceted biological framework involving inflammation, immune response, oxidative stress, microbial interactions, and stress markers. Key findings include the significant presence of Neutrophil extracellular traps (NETs) and higher levels of proinflammatory cytokines and chemokines such as MCP-1, MIP-1α, MIP-1β, CCL-20, RANKL, BAFF, and OPG in peri-implantitis patients. Oxidative stress markers like MDA, TAC, SOD, and GSH-Px, as well as salivary biomarkers such as CRP, TAS, and sAA, indicate the balance between oxidative damage and antioxidant defense mechanisms. MicroRNAs, particularly miRNA-21-3p, miRNA-150-5p, and miR-4484, and extracellular vesicles (EVs) play roles in gene regulation and intercellular communication, respectively. Proteomic and metabolomic markers, including osteonectin and proteins linked with implant loss, provide insights into molecular changes associated with peri-implant tissue breakdown. Microbial markers, such as the salivary microbiome and herpesviruses, underscore the role of microbial communities in disease initiation and progression. Additionally, cortisol and stress markers reflect the body's stress response, which can modulate immune function and inflammation. These integrated findings offer a holistic view of the biological processes underlying peri-implant diseases, facilitating more targeted diagnostic and therapeutic strategies.

Several review studies have investigated the use of biomarkers in peri-implant crevicular fluid (PICF) and salivary samples for the diagnosis and prognosis of peri-implantitis [ 50][51][ 52][53][ 54]. Elevated levels of proinflammatory cytokines, such as interleukin-1β (IL-1β) and interleukin-6 (IL-6), tumor necrosis factor-alpha (TNF-α), and matrix metalloproteinases, have been consistently associated with peri-implantitis based on these review studies [50][ 51][52][ 53][54]. Additionally, alterations in bone loss markers have shown potential as indicators of disease progression and treatment response [50][51][ ref-type="bibr" rid="R52">52][53][ ref-type="bibr" rid="R54">54]. However, the pathology of peri-implantitis is still not fully understood, and there have been recent challenges to the consensus on its aetiology and pathology, especially in comparison with periodontitis [ 54].

Based on findings of our study, we can draw some conclusions about potential pathophysiological pathways of pre- implantitis as below:

### Initial Microbial Colonization and Biofilm Formation

The initial step in the development of peri-implantitis is the colonization of the implant surface by oral microbiota. This includes a diverse range of bacteria (36) and viruses, such as Herpesviruses (34). The biofilm formed by these microorganisms can trigger an inflammatory response in the surrounding tissues. The implant material's physical and chemical properties can influence biofilm formation, which is a precursor to the adaptive behavior of pathogenic bacteria species [55]. Studies have shown that different implant materials, such as titanium and zirconia, can affect the cultivable polymicrobial saliva community and biofilm formation [55][ 56][57].

Activation of Innate Immune Response, Osteoclast Activation and Bone Resorption, and Extracellular Matrix Remodeling

Studies have shown that peri-implantitis is characterized by a more severe inflammatory infiltrate and innate immune response compared to periodontitis [ 58]. The expression of innate immune receptors, such as toll-like receptors (TLRs) and the receptor for advanced glycated end-products (RAGE), is also upregulated in peri-implantitis [58][ 59][60]. Furthermore, research has shown that the innate immune response in peri-implantitis is characterized by a higher influx of innate and adaptive leukocytes to the peri-implant mucosa, accompanied by increased expression levels of pro-inflammatory cytokines [59][60].

Osteoclast activation and bone resorption play a crucial role in the development of peri-implantitis, a bacteria-induced chronic inflammatory process that affects up to 50% of dental implants [61].

The mechanisms of bone loss around dental implants are poorly understood, but humoral factors and bacterial lipopolysaccharides are thought to stimulate osteoclast differentiation and function [62]. The immune system and bone tissue have an intimate relationship, and immune-inflammatory-induced osteoclast differentiation and function are thought to be the major underlying mechanism of uncoupled bone resorption to bone formation in peri-implantitis [63].

### Angiogenesis and Vascular Changes, Oxidative Stress and Antioxidant Defense, and Stress and Hormonal Responses

Compromised vascular density hinders the tissue's ability to combat infection and provide essential nutrients, making angiogenesis, the process of new blood vessel formation, crucial for healing and immune defense [64]. Enhancing angiogenesis in peri-implant soft tissue holds promise for tissue integration and inflammation control [64]. Vascular endothelial growth factor (VEGF) plays a key role in angiogenesis, and its expression has been studied in the context of peri-implant tissues [65]. Oxidative stress plays a significant role in the pathogenesis of peri-implantitis, as the inflammatory response generates reactive oxygen species (ROS) that can damage cellular components and exacerbate inflammation (Mousavi Jazi et al., 35; Song et al., 44). Markers of oxidative stress, such as malondialdehyde (MDA), total antioxidant capacity (TAC), superoxide dismutase (SOD), glutathione peroxidase (GSH-Px), and salivary total antioxidant status (TAS), can be used to assess the level of oxidative stress and antioxidant defense mechanisms in peri-implantitis (Mousavi Jazi et al., [35]; Song et al., [44]; Drafta et al., [10]; López-Jornet, [33]). The antioxidant defense system attempts to mitigate the damage caused by oxidative stress, but elevated levels of cortisol, a stress hormone, can suppress immune function and affect bone metabolism, further contributing to the progression of peri-implantitis (Alresayes et al., [22]; Aldulaijan, [29]; Soysal et al., [45]).

## Conclusion

This review summarizes the existing research on biomarkers linked to peri-implantitis, highlighting their potential as non-invasive methods for early detection, monitoring, and management. It suggests that future research should focus on developing standardized protocols and performing clinical trials to validate the diagnostic precision and clinical importance of these biomarkers. The current shortcoming in the development of diagnostic approaches is a cultural shortcoming that requires an update of the scientific knowledge of dental professionals.

## Conflict of Interest

None.
